# Activation of Water on MnO_x_-Nanocluster-Modified Rutile (110) and Anatase (101) TiO_2_ and the Role of Cation Reduction

**DOI:** 10.3389/fchem.2019.00067

**Published:** 2019-02-12

**Authors:** Stephen Rhatigan, Michael Nolan

**Affiliations:** Tyndall National Institute, University College Cork, Cork, Ireland

**Keywords:** TiO2, water activation, reduction, oxygen vacancies, photocatalysis

## Abstract

Surface modification of titania surfaces with dispersed metal oxide nanoclusters has the potential to enhance photocatalytic activity. These modifications can induce visible light absorption and suppress charge carrier recombination which are vital in improving the efficiency. We have studied heterostructures of Mn_4_O_6_ nanoclusters modifying the TiO_2_ rutile (110) and anatase (101) surfaces using density functional theory (DFT) corrected for on-site Coulomb interactions (DFT + U). Such studies typically focus on the pristine surface, free of the point defects and surface hydroxyls present in real surfaces. In our study we have considered partial hydroxylation of the rutile and anatase surfaces and the role of cation reduction, via oxygen vacancy formation, and how this impacts on a variety of properties governing the photocatalytic performance such as nanocluster adsorption, light absorption, charge separation, and reducibility. Our results indicate that the modifiers adsorb strongly at the surface and that modification extends light absorption into the visible range. MnO_x_-modified titania can show an off-stoichiometric ground state, through oxygen vacancy formation and cation reduction spontaneously, and both modified rutile and anatase are highly reducible with moderate energy costs. Manganese ions are therefore present in a mixture of oxidation states. Photoexcited electrons and holes localize at cluster metal and oxygen sites, respectively. The interaction of water at the modified surfaces depends on the stoichiometry and spontaneous dissociation to surface bound hydroxyls is favored in the presence of oxygen vacancies and reduced metal cations. Comparisons with bare TiO_2_ and other TiO_2_-based photocatalyst materials are presented throughout.

## Introduction

Photocatalysts are semiconductor materials which absorb photons of energies in excess of the bandgap to produce electron-hole pairs. These charge carriers separate and migrate to the surface of the catalyst where they drive chemical reactions *via* reduction and oxidation of adsorbed species. Photocatalysis has a variety of applications, including, but not limited to, the solar production of hydrogen from water splitting (Ni et al., 2007; Fujishima et al., 2008; Maeda and Domen, 2010; Jiang et al., 2017).

A practical photocatalyst must meet a number of criteria, such as visible light absorption, efficient charge carrier separation, stability, and active surface sites for the adsorption of feedstock species. Reducible metal cations in the catalyst can be important for enhancing the activity of the catalyst toward the difficult step of water dissociation. The development of metal oxide photocatalysts is of interest as these materials are cheap, earth abundant and, in many instances, non-toxic. Indeed, the most widely studied photocatalyst is titanium dioxide (TiO_2_) (Ni et al., 2007; Fujishima et al., 2008; Dimitrijevic et al., 2011; Pelaez et al., 2012; Habisreutinger et al., 2013; Tada et al., 2014; Etacheri et al., 2015) which was first demonstrated as a photoanode for water splitting by Fujishima and Honda (1972). The large bandgap (>3 eV) means that photoactivity is restricted to the UV and has limited the real-world application of TiO_2_-based photocatalyst technologies. As a result, significant scientific effort has focused on extending the light absorption edge of TiO_2_ to longer wavelengths.

Substitutional doping of TiO_2_ with cations and/or anions is a widely studied approach to inducing visible light absorption through the emergence of impurity-derived energy levels in the TiO_2_ bandgap (Di Valentin et al., 2007; Czoska et al., 2008; Haowei et al., 2008; Ikeda et al., 2008; Gai et al., 2009; Nie et al., 2009; Valentin et al., 2009; Xu et al., 2009; Yang et al., 2009; Yu et al., 2009; Zhu et al., 2009; Long and English, 2010a,b; Zhang et al., 2010; Zheng et al., 2010; Herrmann, 2012; Etacheri et al., 2015; Li, 2015; Na Phattalung et al., 2017). First principles studies of such doped systems typically focus on bandgap reduction (Cui et al., 2008; Yang et al., 2009; Zhu et al., 2009; Long and English, 2010a,b; Zhang et al., 2010, 2014; Chand et al., 2011; Guo and Du, 2012; Na Phattalung et al., 2017) and questions of charge localization and surface reactivity are often overlooked. These are important considerations as dopant-derived defect states have been shown to act as recombination centers (Herrmann, 2012; Etacheri et al., 2015; Li, 2015) and photocatalysis is generally a surface mediated phenomenon.

Studies of the chemistry and electronic properties of surfaces and interfaces are key to understanding and screening materials for photocatalysis. The enhanced performance of the benchmark material, P25, which consists of chemically interfaced rutile and anatase phases, has been attributed to the favorable alignment of the conduction and valence bands at the interface which facilitates charge transfer between phases and the suppression of charge carrier recombination (Scanlon et al., 2013). In addition, the interface can promote the formation of active catalytic sites. This effect can be tuned by considering heterostructures with metal oxides of different compositions. Such heterostructures have been realized experimentally and shown to exhibit enhanced photocatalytic activity (Boppana and Lobo, 2011; Boppana et al., 2013; Chae et al., 2017; Sotelo-Vazquez et al., 2017; Wang et al., 2017). Nanostructuring of metal oxides has been investigated as an approach to enhancing charge transfer kinetics and increasing surface area while providing low-coordinated metal and oxygen sites for the adsorption of feedstock species (Gordon et al., 2012; Bhatia and Verma, 2017; Zhang et al., 2017a; Ong et al., 2018). Further, nanostructuring can also facilitate the reduction of metal cations.

Surface modification of metal oxide surfaces with dispersed metal oxide nanoclusters combines the properties of hetero- and nano-structuring. Sub-nm nanoclusters of iron oxide were deposited on TiO_2_ surfaces *via* chemisorption-calcination cycle (CCC) (Jin et al., 2011; Tada et al., 2014) and atomic layer deposition (ALD) (Libera et al., 2010). FeO_x_-modified TiO_2_ exhibited bandgap reduction and enhanced visible light photocatalytic activity. The modification was shown to suppress carrier recombination as indicated by photoluminescence spectroscopy (Jin et al., 2011). The red-shift in light absorption was attributed to cluster-derived states above the valence band maximum (VBM) which were identified by X-ray photoelectron spectroscopy (XPS) and density functional theory (DFT) simulations (Jin et al., 2011; Nolan, 2011a; Tada et al., 2014).

These studies, and the subsequent development of similar systems, (Jin et al., 2012; Boppana et al., 2013; Iwaszuk et al., 2013; Bhachu et al., 2014; Fronzi et al., 2016a) mean that a multitude of nanocluster-surface composites can be investigated. Considerations for tuning these systems for optimal performance include composition, surface termination, nanocluster size, and stoichiometry; all of which contribute to the light absorption properties, charge carrier mobility and surface reactivity. DFT simulations can be used to illuminate the properties underpinning experimental observations (Nolan, 2011a; Jin et al., 2012; Nolan et al., 2012; Iwaszuk et al., 2013) and to screen candidate materials worthy of further investigation (Park et al., 2009; Graciani et al., 2010; Nolan, 2011b, 2012, 2018; Iwaszuk and Nolan, 2013; Lucid et al., 2014; Nolan et al., 2014; Fronzi et al., 2016b; Rhatigan and Nolan, 2018a,b).

In the present study we use first principles DFT calculations to examine the photocatalytic properties of manganese oxide modified TiO_2_, using model systems of Mn_4_O_6_-nanoclusters modifying the rutile (110) and anatase (101) surfaces and consider the role of partial surface hydroxylation in the interfacial chemistry. Our analysis includes an assessment of the stability of the composite surfaces, their ground state stoichiometry and reducibility *via* oxygen vacancy formation. Point defects, such as oxygen vacancies, are active sites at metal oxide surfaces and can be produced thermally. A more reducible surface will lose oxygen more readily and be more active in solar thermal (Muhich et al., 2016) or Mars and van Krevelen processes 1954, (Ganduglia-Pirovano et al., 2007). Computed density of states plots elucidate the impact of modification on the light absorption properties and a model for the photoexcited state (Di Valentin and Selloni, 2011) is used to examine charge separation and localization. Finally, we study the interaction of water with the modified surfaces and focus particularly on the role of oxygen vacancies and reduced cations on water adsorption. We identify the characteristics of activation, such as dissociation, geometry distortions and charge transfer to the adsorbed species. The importance of oxygen vacancies as active sites for water dissociation at the rutile (110) surface (Schaub et al., 2001; Henderson et al., 2003) and ceria surfaces (Mullins et al., 2012) has been widely discussed and reduced Ti^3+^ ions have been shown to be active in the chemistry at titania surfaces (Lira et al., 2011; Xiong et al., 2012). For anatase TiO_2_, oxygen vacancies have been shown to be more stable at subsurface and bulk sites than on the surface (He et al., 2009; Scheiber et al., 2012). However the surface can be reduced by electron bombardment (Scheiber et al., 2012; Setvin et al., 2016) and the reaction of these vacancy sites with water and O_2_ results in water dissociation. These studies highlight the necessity of engineering photocatalytic surfaces for which vacancies can be produced with moderate energy costs.

MnO_x_ is an interesting modifier as manganese is a multi-valent, reducible element which crystallizes in oxides with a variety of oxidation states; (Franchini et al., 2007) this will have implications for the light absorption properties and reducibility of sub-nm nanoclusters of MnO_x_ dispersed at the titania surfaces. We have previously studied similar systems of MnO_x_-modified TiO_2_, in collaboration with experiment, to interrogate their activity for CO_2_ capture and reduction (Schwartzenberg et al., 2017). In the present study, we focus on the potential for these catalysts to be active toward water activation. Furthermore, we investigate the impact of surface hydroxylation on the reduction of the heterostructures *via* oxygen vacancy formation and apply a model for photoexcitation to examine the associated energetics and charge localization. In reference Schwartzenberg et al. (2017), the Mn_4_O_6_-TiO_2_ composites were found to be stoichiometric in the ground state for both modified rutile and anatase, albeit with moderate costs to produce reducing oxygen vacancies (+0.59 eV for rutile and +1.1 eV for anatase). However, the impact of surface hydroxyls on the formation of oxygen vacancies was not investigated; in this paper we show that vacancy formation is in fact promoted with hydroxyls already present at the TiO_2_ surfaces. The photoexcited state model, which examines localization of electrons and holes at nanocluster metal and oxygen sites, sheds light on experimental observations which suggest that the MnO_x_-modifiers may facilitate recombination (Schwartzenberg et al., 2017). In addition, active oxygen vacancy sites play a crucial role in the subsequent interaction of water molecules and their adsorption modes. In particular, dissociation is favored for the reduced systems; this is an important step in the water oxidation reaction.

## Methodology

Periodic plane wave DFT calculations are performed using the VASP5.4 code (Kresse and Hafner, 1994; Furthmüller et al., 1996) with an energy cut-off of 400 eV. The core-valence interaction is described with projector augmented wave (PAW) potentials, (Blöchl, 1994; Kresse and Joubert, 1999) with 4 valence electrons for Ti, 6 for O, 13 for Mn and 1 for H species. The Perdew-Wang (PW91) approximation to the exchange-correlation functional is used (Perdew et al., 1996).

The TiO_2_ rutile (110) and anatase (101) substrates are modeled as 18 and 12 atomic layer slabs, respectively. The bulk lattice parameters for rutile were computed as a = 4.639 Å and c = 2.974 Å, and the rutile (110) surface was modeled with a (2×4) surface expansion. For anatase the bulk lattice parameters are a = 3.814 Å and c = 9.581 Å and a (1 × 4) expansion was used for the anatase (101) surface. These parameters correspond to surface areas per supercell of 13.120 × 11.896 Å and 10.312 × 15.255 Å for rutile (110) and anatase (101), respectively. The surfaces are separated from their periodic images by vacuum gaps of 20 Å, as used in our previous studies (Fronzi and Nolan, 2017; Schwartzenberg et al., 2017; Nolan, 2018; Rhatigan and Nolan, 2018a,b). Γ-point sampling is used and the convergence criteria for the energy and forces are 10^−4^ eV and 0.02 eV^−2^, respectively. All calculations are spin polarized.

A Hubbard U correction is implemented to consistently describe the partially filled Mn 3d states and reduced Ti^3+^ states (Anisimov et al., 1991; Dudarev et al., 1998). The values of U used are U(Ti) = 4.5 eV and U(Mn) = 4 eV and these have been chosen based on previous work on TiO_2_ (Morgan and Watson, 2007; Nolan et al., 2008; Iwaszuk and Nolan, 2011; Nolan, 2011a; Fronzi et al., 2016a; Fronzi and Nolan, 2017; Rhatigan and Nolan, 2018a) and manganese oxides (Franchini et al., 2007; Kitchaev et al., 2016).

To model surface hydroxylation (before the nanoclusters are adsorbed) and the impact on the heterostructure chemistry, four water molecules are dissociatively adsorbed at the clean rutile (110) and anatase (101) surfaces which gives a partial coverage of 50%. The computed energy gain when the TiO_2_ surfaces are hydroxylated at half coverage is −1.03 eV per water molecule for rutile (110) and −0.8 eV for anatase (101), referenced to the total energy of four gas phase water molecules. These indicate favorable water adsorption and surface hydroxylation and these models have been used in our previous studies (Fronzi et al., 2016a; Fronzi and Nolan, 2017; Schwartzenberg et al., 2017; Rhatigan and Nolan, 2018a). The nature of water molecules adsorbed at metal oxide surfaces, and in particular TiO_2_ surfaces, is widely investigated both experimentally and computationally (Valdés et al., 2008; Fronzi and Nolan, 2017; Rhatigan and Nolan, 2018a) and readers are referred to reference (Mu et al., 2017) for a review of the state of the art. These models are representative of hydroxylated rutile and anatase surfaces, while we are not attempting to describe the most stable solutions for water or dissociative water adsorption at these titania surfaces (Fronzi et al., 2016a; Fronzi and Nolan, 2017; Rhatigan and Nolan, 2018a). The hydroxylated surfaces are denoted by OH-r110 and OH-a101. For the O^2−^ ions of the pristine titania surfaces, computed Bader charges are in the range of 7.3–7.4 electrons and this is our reference. After hydroxylation, Bader charges for those oxygen atoms of the surface to which H atoms are adsorbed increase to values in the range 7.6–7.7 electrons, with similar values for oxygen ions of the water-derived hydroxyls.

The Mn_4_O_6_ nanocluster (see Supporting Information) was adsorbed in different configurations at the hydroxylated rutile (110) and anatase (101) surfaces and the adsorption energies are computed using:

(1) $$\begin{array}{r} E_{ads}=E_{surf+A}-E_{surf}-E_{A} \end{array}$$

where *E*_*surf*+*A*_, *E*_*surf*_ and *E*_*A*_ are the energies of the adsorbate-surface composite system, the hydroxylated titania surface and the gas phase nanocluster, respectively.

For the reduction of the composite surface, each of the six O sites of the supported nanocluster is considered for the formation of an oxygen vacancy, O_V_. One oxygen ion is removed from the Mn_4_O_x_ cluster and the vacancy formation energy is calculated as:

(2) $$E_{vac}=\;E\left(Mn_{4}O_{x-1}\right)+1/2E(O_{2})-E\left(Mn_{4}O_{x}\right)$$

where the first and third terms on the right hand side are the total energy of the cluster-surface composite with and without an oxygen vacancy and the energy is referenced to half the total energy for molecular O_2_. Having identified the most stable structure with a single O_V_, the calculation is repeated for each of the five remaining O sites to determine the most stable structure with two O_V_. Oxidation states are investigated with Bader charge analysis and computed spin magnetizations.

We apply a model for photoexcitation to the ground state configuration of each modified surface and to the unmodified OH-r110 and OH-a101 surfaces for comparison. This model involves imposing a triplet electronic state on the system (Di Valentin and Selloni, 2011) to promote an electron to the conduction band, with a corresponding hole in the valence band. The analysis of the energies and charge localization is discussed in more detail in the Supporting Information.

For the interaction of water with the modified surfaces, H_2_O molecules are adsorbed in various configurations at the oxygen deficient systems and the adsorption energies are calculated as:

(3) $$\begin{array}{r} E_{ads}=E_{surf+H_{2}O}-E_{surf}-E_{H_{2}O} \end{array}$$

where *E*_*surf*+*H*_2_*O*_, *E*_*surf*_ and *E*_*H*_2_*O*_ refer to the energies of the H_2_O molecule and modified surface in interaction, the modified surface, and the gas phase H_2_O, respectively.

Oxygen atoms of the surface, cluster and surface-bound hydroxyls are denoted O_S_, O_C_ and O_OH_, respectively, and similar notation is adopted for OH groups. For the interaction of water with the modified surfaces, water-derived oxygen and hydroxyls are denoted O_W_ and OH_W_.

## Results

### Stoichiometric Mn_4_O_6_-Modified TiO_2_ OH-Rutile (110) and OH-Anatase (101)

**Figure 1:**
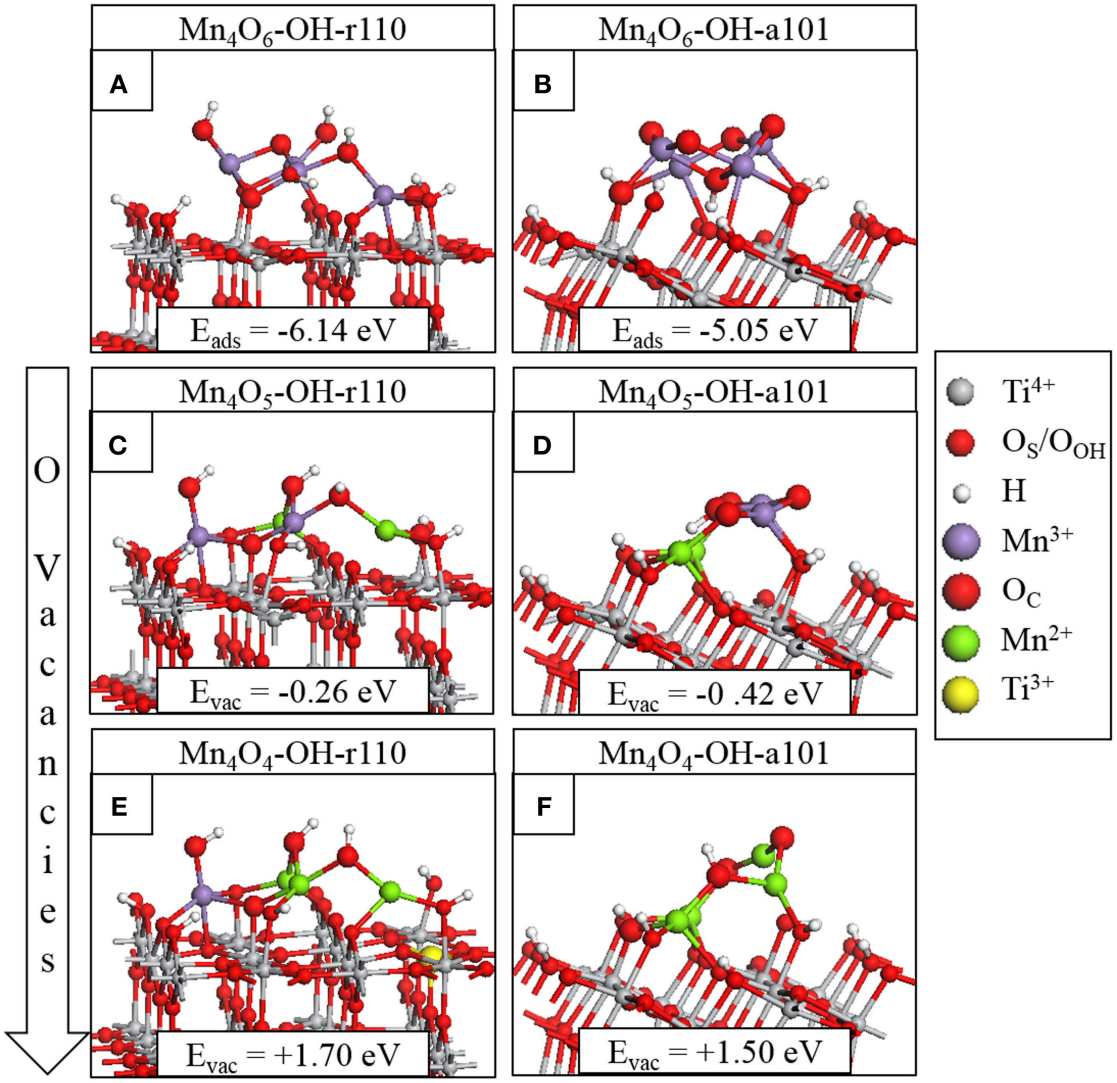
Relaxed atomic structures of Mn_4_O_x_ modifying the hydroxylated titania surfaces. The stoichiometric composites are shown in **(A)** for Mn_4_O_6_-OH-r110 and **(B)** for Mn_4_O_6_-OH-a101; the nanocluster adsorption energies are included in the inset. The atomic structures after formation of the most stable single O_V_ are shown in panels **(C)** for Mn_4_O_5_-OH-r110 and **(D)** for Mn_4_O_5_-OH-a101. The atomic structures of the most stable composites with two O_V_ are shown in panels **(E)** for Mn_4_O_4_-OH-r110 and **(F)** for Mn_4_O_4_-OH-a101. The energy costs to produce O_V_ are included and computed relative to the structure with one less O_V_. Atomic species and oxidation states are indicated by the colors in the legend on the right hand side.

Figures 1A,B show the adsorption energies and relaxed atomic structures of the stoichiometric Mn_4_O_6_-nanocluster modifying the OH-r110 and OH-a101 surfaces. The large, negative adsorption energies indicate that the nanocluster-surface interaction is favorable and that the nanoclusters will be stable against desorption and aggregation (Fronzi et al., 2016b; Nolan et al., 2016; Fronzi and Nolan, 2017; Nolan, 2018; Rhatigan and Nolan, 2018a,b). For Mn_4_O_6_-OH-r110 (Figure 1A), three Mn ions are 4-fold coordinated and to each of these is bound a terminal OH. Of these OH groups, one has migrated from a Ti site in the rutile surface to an Mn ion of the cluster (OH_OH_) and two OH groups result from the migration of hydrogen from surface hydroxyls to O_C_ atoms (OH_C_). The fourth Mn ion is 5-fold coordinated and is bound to three O_C_ and two O_S_ ions (one bridging O_S_ and one in-plane O_S_). Five O ions of the OH-r110 surface bind with Mn of the nanocluster (three O_S_ and two O_OH_) and two O_C_ ions bind to Ti of the surface. Mn-O distances are in the range 1.8–2.1 Å; the shorter distances involve 2-fold coordinated O ions and for Mn bound to the in-plane O_S_ ion the Mn-O distance is 2.2 Å. Ti ions which bind to the nanocluster migrate out from the surface by 0.1 Å, however, distortions to the geometry of the rutile (110) surface are minimal.

For Mn_4_O_6_-OH-a101 (Figure 1B), three Mn ions are 4-fold coordinated and one Mn is 5-fold coordinated. Five O_C_ sites are 2-fold coordinated with one O_C_ ion binding to three Mn ions and a H atom which has migrated from a bridging O_S_ site. Of the six interfacial bonds between the Mn_4_O_6_ nanocluster and OH-a101, three involve Mn and OH_OH_ groups; two involve Mn and bridging O_S_ sites and the sixth is a Ti-O_C_ bond. Mn-O distances are in the range 1.7–2.1 Å.

For Mn_4_O_6_ adsorbed at OH-r110 and OH-a101, the computed Bader charge for each of the Mn ions is 11.3 electrons, which are typical of Mn^3+^ ions (see Table 1).(Schwartzenberg et al., 2017) The spin magnetizations for these sites are each 3.9 μ_B_, which reflects the 3d^4^ configuration of the Mn^3+^ ion_._

**Table 1:** Computed Bader charges for the manganese ions in the supported nanoclusters before and after formation of one or more O_V_.

**Surface**	**OH-r110**				**OH-a101**		
**Modifier**	**Mn_**4**_O_**6**_**	**Mn_**4**_O_**5**_**	**Mn_**4**_O_**4**_**		**Mn_**4**_O_**6**_**	**Mn_**4**_O_**5**_**	**Mn_**4**_O_**4**_**
Mn_I_	11.3	**11.5**	**11.5**	Mn_I_	11.3	**11.5**	**11.5**
Mn_II_	11.3	11.2	11.2	Mn_II_	11.3	11.3	**11.6**
Mn_III_	11.3	11.2	**11.5**	Mn_III_	11.3	11.3	**11.6**
Mn_IV_	11.3	**11.5**	**11.5**	Mn_IV_	11.3	**11.5**	**11.5**
Ti_I_	1.3	1.3	**1.7**				

*Also included are Bader charges for titanium ions of the support which are reduced after vacancy formation. Reduced Mn^2+^ and Ti^3+^ are highlighted in bold*.

For the Mn_4_O_6_ nanocluster adsorbed at OH-a101, there is an accumulation of positive charge at those O_C_ sites which are doubly-coordinated to Mn ions of the nanocluster. Computed Bader charges of 7.0 electrons for these O_C_ sites compare with 7.3–7.7 electrons computed for O^2−^ anions of the OH-a101 surface. The nanocluster-surface interaction is not as strong at the OH-a101 surface as indicated by the smaller adsorption energy. The consequence of this is that the supported nanocluster retains characteristics of the gas phase, for which the O_C_ ions have computed Bader charges in the range 7.0–7.1 electrons.

### Reduction of Mn_4_O_6_-Modified TiO_2_ OH-Rutile (110) and OH-Anatase (101) Via Oxygen Vacancy Formation

The most stable modified surfaces with a single O_V_ are shown in Figure 1C for Mn_4_O_5_-OH-r110 and Figure 1D for Mn_4_O_5_-OH-a101. For the modified OH-r110 surface the formation energy of a single O_V_ is −0.26 eV and this formation energy indicates that O_V_ will form spontaneously. The next three most stable vacancy sites have formation energies in the range 0.60–0.82 eV. After formation of the most stable vacancy, two Mn ions are 3-fold coordinated and the third and fourth Mn cations are 4- and 5-fold coordinated. Two bridging and one in-plane surface oxygen are bound to Mn ions of the nanocluster. Two O_C_ ions bind to surface Ti sites while three O_C_ ions are bound only to Mn and H ions.

The formation of the neutral oxygen vacancy releases two electrons. Bader charge analysis reveals that the electrons localize at the 3-fold coordinated Mn sites of the nanocluster. The computed Bader charges on these sites increase from 11.3 to 11.5 electrons; see (Table 1) for computed Bader charges of reduced Ti and all Mn sites. The computed spin magnetizations are 4.6 μ_B_ for these Mn sites; this is typical of the formation of reduced Mn^2+^ ions which has an electronic configuration of 3d^5^.

The most favorable structure with one O_V_ is more stable than the second most favorable by 0.9 eV. However, the relaxed atomic structures of these configurations are very similar (compare Figure 1C with Figure S3A). The difference in energy arises from the distibution of excess charge. For the O_V_ structure shown in Figure S3A, one excess charge localizes at a 5-fold coordinated surface Ti site for which the Bader charge increases from 1.3 to 1.7 electrons. A computed spin magnetization of 1.0 μ_B_ reflects the 3d^1^ configuration of reduced Ti^3+^.

For the modified OH-a101 surface, the most stable O_V_ has a formation energy of −0.42 eV which indicates that it will spontaneously form, so that the ground state is off-stoichiometric (vacancy formation energies for other sites of the nanocluster were in the range 0.5–1.3 eV). This compares with Mn_4_O_6_ modifying bare anatase (101) which was found to be stoichiometric in the ground state (Schwartzenberg et al., 2017). After the formation of this O_V_, two Mn ions relax toward the titania surface and bind with bridging O_S_ sites so that, in this configuration, each Mn ion is 4-fold coordinated. Bader charge analysis and computed spin densities indicate that two Mn ions are reduced to Mn^2+^, having computed Bader charges of 11.5 electrons and computed spin magnetisations of 4.6 μ_B_. The next most stable structure with one O_V_ is shown in Figure S3B; in this configuration three Mn ions are reduced to Mn^2+^ and this is accompanied by an accumulation of postive charge on 2-fold coordinated O_C_ ions for which the Bader charges were computed as 7.0 electrons.

The formation of the second O_V_ has a moderate energy cost for both MnO_x_-modified TiO_2_ surfaces, however the modified anatase surface is reducible at a lower energy cost. Given that the anatase surface is more easily hydroxylated, (Mu et al., 2017) which these results indicate promotes vacancy formation, one would expect more O_V_ present on modified anatase. That O_V_ formation is more facile for modified anatase corroborates previous experimental work on MnO_x_-TiO_2_ (Schwartzenberg et al., 2017). The most stable configurations of the heterostructures with two O_V_ are shown in Figure 1E for Mn_4_O_4_-OH-r110 and Figure 1F for Mn_4_O_4_-OH-a101. For the Mn_4_O_4_-OH-r110 surface, the two most stable O_C_ sites for formation of a second O_V_ had similar formation energies. One such configuration is described here and the other is included in the Supporting Information. For the structure shown in Figure 1E, the removed O_C_ ion was 2-fold coordinated to a cluster Mn and surface Ti ion. After vacancy formation the Mn ion binds to a bridging O_S_ ion and remains 3-fold coordinated. In this configuration three Mn ions are reduced; the Bader charges and spin magnetizations for these sites are 11.5 electrons and 4.6 μ_B_, respectively. Similarly, for the Ti site to which the removed O_C_ was bound, the Bader charge and spin magnetization are 1.7 electrons and 1.0 μ_B_. Hence, the Mn_4_O_4_-OH-r110 heterostructure with two oxygen vacancies has one Ti^3+^ and three Mn^2+^ ions.

For the modified OH-a101 surface, a 3-fold coordinated O_C_ site, which forms a hydroxyl group bridging two Mn ions, has the lowest cost to produce a second O_V_. One Mn ion that was bound to the removed O_C_ atom is 2-fold coordinated, having been originally coordinated to three O_C_ ions and one O_OH_ ion. The second Mn ion is 3-fold coordinated, having been 4-fold coordinated prior to vacancy formation. The H ion which was bound to the removed O_C_ migrates to another O_C_ ion. In this Mn_4_O_4_-OH-a101 configuration, there are four Mn^2+^ ions, with computed Bader charges of 11.5–11.6 electrons and spin magnetizations of 4.6 μ_B_.

Additional structures with two O_V_ are presented in Figure S3C for Mn_4_O_4_-OH-r110 and Figure S3D for Mn_4_O_4_-OH-a101; these are close in energy to the configurations described above, and differ in the distribution of excess charge over Mn and Ti sites. Hence, Mn and Ti sites should be present at the surface in a variety of oxidation states.

The localization of electrons at Ti and Mn sites is also accompanied by localized geometry distortions. The cation-O distances increase by ~0.1 Å after reduction, reflecting the larger ionic radii of Mn^2+^ and Ti^3+^ compared to Mn^3+^and Ti^4+^ (Shannon and Prewitt, 1969).

### Electronic Properties of Mn_4_O_x_-Modified TiO_2_ OH-Rutile (110) and OH-Anatase (101)

**Figure 2:**
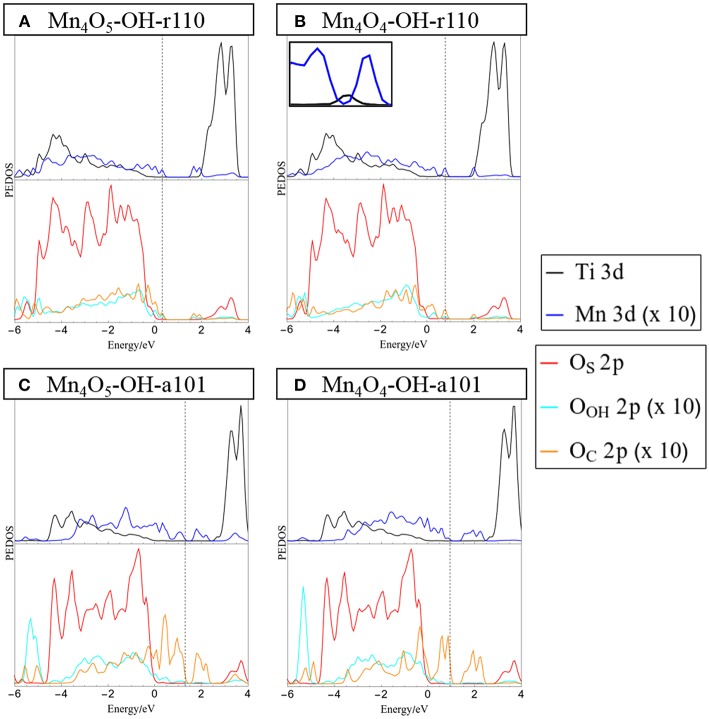
Projected electronic density of states (PEDOS) plots for **(A)** Mn_4_O_5_-, and **(B)** Mn_4_O_4_-OH-r110 and **(C)** Mn_4_O_5_-, and **(D)** Mn_4_O_4_-OH-a101. The computed valence band max is set to 0 eV and the Fermi energy is indicated with a dashed line. The top half of each panel displays Ti and Mn 3d derived states. The bottom halves of the panels show contributions to the DOS from oxygen 2p states of the surface (O_C_), surface bound hydroxyls (O_OH_) and nanocluster (O_C_). Inset in panel **(B)** shows the mid-gap occupied Ti 3d states in the range [0 eV, 1 eV].

The projected electronic density of states (PEDOS) for the heterostructures are presented in Figure 2. Since the heterostructures are off-stoichiometric in the ground state, the PEDOS plot for Mn_4_O_6_-OH-r110 and Mn_4_O_6_-OH-a101 have been omitted from this figure and are included in the Supporting Information for completeness. The top panels of Figure 2 show the PEDOS of modified OH-r110 for (A) the ground state with one O_V_ and (B) the reduced state with two O_V_. The PEDOS plots show that occupied nanocluster-derived states (Mn 3d and O_C_ 2p) extend to 0.3 and 0.8 eV above the VBM of the rutile support for Mn_4_O_5_- and Mn_4_O_4_-OH-r110, respectively. Unoccupied Mn 3d-derived states also emerge in the titania band gap at 0.1 and 0.3 eV below the conduction band minimum (CBM) for the ground state with one O_V_ and the reduced state with two O_V_. Additional states emerge in the band gap due to occupied Ti^3+^ states (see inset of Figure 2B), for the heterostructure with two O_V_.

The bottom panels of Figure 2 display the PEDOS of the modified OH-a101 surface for (C) the ground state, with one O_V_, and (D) reduced state with two O_V_. The PEDOS plot for the ground state, with one O_V_, shows that occupied Mn 3d- and O_C_ 2p-derived states extend to 1.3 eV above the titania derived VBM, while unoccupied Mn 3d states emerge at 1 eV below the CBM, leading to a significant reduction in the computed energy gap relative to TiO_2_. For the reduced structure, with two O_V_, each of the Mn ions is reduced to Mn^2+^, and the highest occupied of these states is 1 eV above the VBM. The lowest energy, unoccupied state is Mn-derived and is 1 eV below the CBM. For Mn_4_O_4_-OH-a101 the energy gap is 0.6 eV, with our DFT+U set-up showing a reduction over unmodified anatase.

These features of the PEDOS for Mn_4_O_x_-TiO_2_ can be attributed to formation of interfacial bonds, the presence of low-coordinated Mn and O_C_ sites and the facile formation of O_V_ in the supported metal oxide nanocluster. Modification pushes the VBM to higher energy and results in the emergence of empty states below the CBM; these effects, and the consequent red shift, are greater for modified anatase, consistent with previous reports (Schwartzenberg et al., 2017). These metal oxide nanocluster-modified surfaces are of interest for the oxygen evolution half reaction (OER) of the water splitting process and in this context raising the VBM from that of TiO_2_ and toward the water oxidation potential is a desirable effect. Lowering of the titania CBM from its favorable position straddling the water reduction potential is detrimental to the hydrogen evolution reaction (HER) activity. However, as H adsorbs too strongly at metal oxide surfaces, such heterostructures will in any case not be suitable photocathodes for water splitting.

### Photoexcitation Model

We apply the model for the photoexcited state to the ground state systems, Mn_4_O_5_-OH-r110 and Mn_4_O_5_-OH-a101. Table 2 presents the computed vertical, singlet-triplet and electron-hole trapping energies, as discussed in the Supporting Information. As can be seen from the values listed in Table 2, the underestimation of the bandgap inherent in approximate DFT is present in our DFT+U computational set-up. Our goal in choosing +U corrections is to consistently describe the localization of electrons and holes rather than reproduce the bandgap of bulk TiO_2_, which is not advised. Comparison of these computed energies across different structures nonetheless yields useful qualitative information about the effect of surface modification and results for the unmodified OH-r110 and OH-a101 surfaces are included for reference. In particular, E^vertical^ is analogous to the optical band gap, and a reduction in this value for a heterostructure relative to unmodified titania implies that modification leads to a red shift in light absorption.

**Table 2:** Vertical singlet-triplet energy difference (E^vertical^), the relaxed singlet-triplet energy difference (E^excite^) and the relaxation energy (E^relax^) for Mn_4_O_5_-OH-r110 and Mn_4_O_5_-OH-a101.

**Composite structure**	**E^**vertical**^ (eV)**	**E^**excite**^ (eV)**	**E^**relax**^ (eV)**
OH-rutile (110)	2.08	1.61	0.46
Mn_4_O_5_-OH-rutile (110)	2.00	0.68	1.31
OH-anatase (101)	2.71	1.52	1.19
Mn_4_O-_5_-OH-anatase (101)	2.37	0.95	1.43

*Values for hydroxylated rutile (110) and anatase (101) surfaces have been included for reference*.

When comparing Mn_4_O_5_-OH-r110 with unmodified OH-r110 we can see that the values for E^vertical^ are similar, however E^excite^ is reduced by 0.93 eV for the modified surface. Comparing Mn_4_O_5_-OH-a101 with unmodified OH-a101, decreases in E^vertical^ and E^excite^ by 0.34 and 0.57 eV, respectively, indicate that modification leads to a significant red shift in light absorption. These results corroborate the analysis of the PEDOS. E^relax^ is the energy gained by the system after structural relaxation in response to the triplet electronic state and is related to the stability of the trapped electron and hole. The relaxation energy is larger for Mn_4_O_5_-OH-r110 than that computed for unmodified OH-r110 (1.31 vs. 0.46 eV) and reflects the greater flexibility of the modified system in accommodating the triplet electronic state. The relaxation energies for Mn_4_O_5_-OH-a101 and unmodified OH-a101 are comparable. The mixture of Mn oxidation states and the proximity of the Mn ions to each other at the anatase surface (neighboring Mn-Mn distances are in the range 2.9–3.2 Å for Mn_4_O_5_-OH-a101 and 3.0–3.9 Å for Mn_4_O_5_-OH-r110) restricts the degree to which the nanocluster can respond structurally to the localization of photoexcited charges.

**Figure 3:**
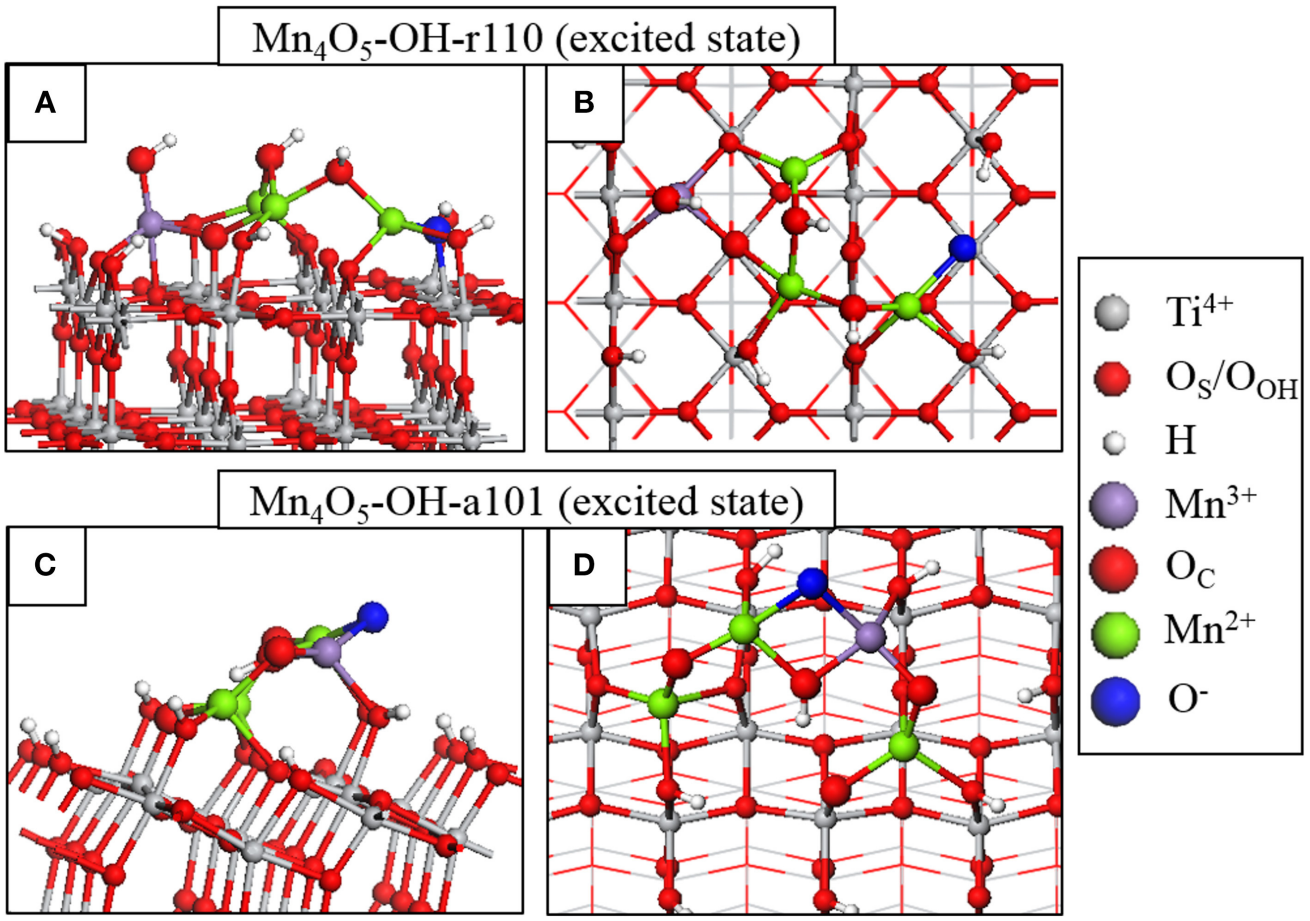
Atomic structure of the fully relaxed triplet electronic state imposed on Mn_4_O_6_-OH-r110 for **(A)** side and **(B)** top view and Mn_4_O_5_-OH-a101 for **(C)** side and **(D)** top view. Charge localization and changes in oxidation state are distinguished by color according to the legend on the right hand side.

Through analysis of Bader charges and spin magnetizations we can determine the electron and hole localization sites and the results of this analysis are represented graphically in Figure 3. For Mn_4_O_5_-OH-r110, in Figures 3A,B, the electron localizes at an Mn site; the Bader charge and spin magnetization for this site are 11.5 electrons and 4.6 μ_B_ after electron localization, which are typical of Mn^2+^ formation. The hole localizes at an O_C_ site which is 2-fold coordinated to the Mn^2+^ ion and a surface Ti. In this instance the Bader charge is 6.8 electrons and the spin magnetization is 0.8 μ_B_, which are consistent with formation of O^−^. The Mn^2+^-O^−^ distance increases by 0.2 Å, relative to the ground state. The Ti-O^−^ distance decreases by 0.1 Å.

For Mn_4_O_5_-OH-a101 (Figures 3C,D), the photoexcited electron localizes at an Mn site of the nanocluster, as confirmed by a computed Bader charge of 11.5 electrons and spin magnetization of 4.5 μ_B_. The hole state localizes predominantly at an O_C_ site which bridges Mn^2+^ and Mn^3+^ ions. After hole localization the Bader charge for the O^−^ ion is 6.7 electrons and the spin magnetization is 0.8 μ_B_. The Mn^2+^-O distances increase by 0.2–0.3 Å.

These results show that the electron localizes at an Mn site of the supported nanocluster and the hole state localizes at a neighboring O_C_ site. Based on this model for the photoexcited state, we can conclude that modification does not necessarily promote the spatial separation of photoexcited charges. However, both electrons and holes will be available at the modified surface for transfer to adsorbed species.

### H_2_O Adsorption at Mn_4_O_x_-Modified OH-Rutile (110) and OH-Anatase (101)

**Figure 4:**
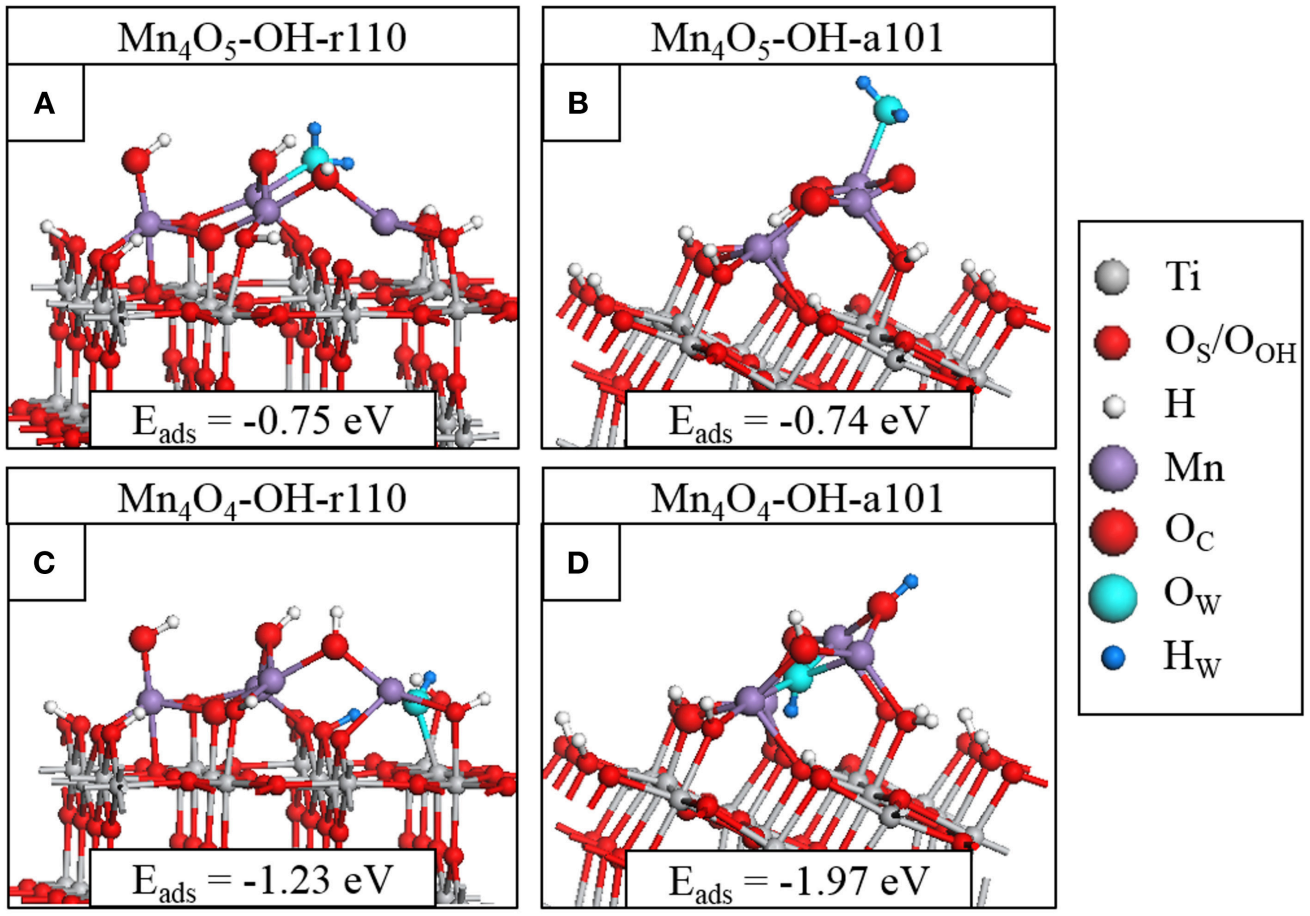
Relaxed atomic structures of the most stable configurations of H_2_O adsorbed at **(A)** Mn_4_O_5_-OH-r110, **(B)** Mn_4_O_5_-OH-a101, **(C)** Mn_4_O_4_-OH-r110, and **(D)** Mn_4_O_4_-OH-a101. Atomic species are distinguished by color according to the legend on the right hand side.

For the interaction of water at the modified surfaces, only those composites with O_V_ present were considered, as such vacancies are known to be active sites at metal oxide surfaces (Schaub et al., 2001; Wang et al., 2016; Ruiz Puigdollers et al., 2017; Zhang et al., 2017b). Water adsorption is favorable at multiple sites of both modified surfaces and the geometries of the most stable adsorption configurations are displayed in Figure 4, while the Supporting Information shows other, less stable, water adsorption structures.

We adsorb water in molecular form at the heterostructures and relax the geometry. For Mn_4_O_5_-OH-r110, shown in Figure 4A, water is adsorbed exothermically in molecular form with a computed adsorption energy of −0.75 eV. In this instance, the water molecule binds to a 3-fold coordinated Mn^2+^ site with a Mn-O_W_ distance of 2.3 Å. Figure S5A shows dissociative water adsorption at the Mn_4_O_5_-OH-r110 surface, which has an adsorption energy of −0.31 eV. Upon dissociation, an H atom migrates to a bridging O_S_ site and the water-derived hydroxyl (OH_W_) is singly coordinated to an Mn site with an Mn-O_W_ distance of 1.9 Å. The dissociation is accompanied by a transfer of charge from O_W_ to the nanocluster modifier, indicated by a decrease of 0.4 electrons in the computed Bader charge for the O_W_ ion. The Bader charges and spin magnetizations of cation sites are unchanged by the adsorption and dissociation.

Water adsorbs molecularly at Mn_4_O_5_-OH-a101, as shown in Figure 4B, with an adsorption energy of −0.74 eV. The H_2_O binds to a 4-fold coordinated Mn^3+^ ion with a Mn-O_W_ distance of 2.2 Å. Since Mn_4_O_5_-OH-r110 and Mn_4_O_5_-OH-a101 are the ground states of the systems, the single O_V_ having formed spontaneously, these composites favor non-stoichiometry so that the strength of interaction with the water molecule is not sufficient to promote spontaneous dissociation and adsorption in molecular form is favored.

The surfaces with two O_V_ show higher reactivity to water, as indicated by the larger adsorption energies in Figures 4C,D. Water adsorbs and spontaneously dissociates at both Mn_4_O_4_-OH-r110 (Figure 4C) and Mn_4_O_4_-OH-a101 (Figure 4D). For Mn_4_O_4_-OH-r110, the water molecule adsorbs at an O_V_ site. An H atoms migrates to a bridging O_S_ site and the OH_W_ group is doubly coordinated to an Mn and a surface Ti site. The Mn-O_W_ and Ti-O_W_ distances are 2.2 Å. Bader charge analysis reveals that 0.3 electrons are transferred from the O_W_ to the surface. Despite this charge transfer, the Ti ion which binds to OH_W_ and which was reduced to Ti^3+^ due to O_V_ formation prior to water adsorption, remains in the Ti^3+^ state. This agrees with work by Henderson et al. in which no charge transfer was observed between Ti^3+^ and bridging hydroxyls bound at oxygen vacancy sites at the TiO_2_ rutile (110) surface (Henderson et al., 2003). The reduced Ti site was only oxidized after interaction of O_2_ with the Ti^3+^-OH group. After water adsorption and dissociation the distribution of cation oxidation states is unchanged so that there are three Mn^2+^ ions and one Ti^3+^. The Bader charge for the bridging O_S_ site to which the H ion binds increases from 7.3 to 7.7 electrons which, as discussed in the methodology, is typical of hydroxyl formation.

For Mn_4_O_4_-OH-a101, the water molecule adsorbs at an O_V_ site and after dissociation an H atom migrates to an O_C_ ion which shows an increase in Bader charge, from 7.1 to 7.6 electrons. The OH_W_ group binds to three Mn^2+^ ions; the Bader charges and spin magnetizations for cation sites are unchanged so that these ions are not involved in the charge transfer. However, for the water adsorption configuration shown in Figure S5D, an Mn^2+^ ion is re-oxidized to Mn^3+^ after dissociation of the water molecule. In this instance the adsorption energy is −1.89 eV and the OH_W_ group is singly-coordinated to the re-oxidized Mn ion.

## Conclusions

The properties of Mn_4_O_x_-modified hydroxylated titania surfaces and their reduction and interaction with water depend on the phase of the TiO_2_ substrate. For Mn_4_O_6_ adsorbed at the hydroxylated anatase (101) surface, one interfacial bond is established between a cluster oxygen ion and the surface and Mn ions bind mostly to oxygen ions of the surface bound hydroxyls. Conversely, for Mn_4_O_6_ at hydroxylated rutile (110), the nanocluster-surface interaction is more intimate, with Mn ions binding to bridging and in-plane oxygen ions of the rutile surface.

Our results indicate that both Mn_4_O_x_-OH-r110 and Mn_4_O_x_-OH-a101 favor non-stoichiometry, in contrast to unhydroxylated modified TiO_2_ surfaces, as oxygen vacancies form spontaneously and both composites can be considered highly reducible with moderate energy costs for subsequent oxygen vacancy formation. Bader charge analysis shows that Mn ions are present in a mixture of oxidation states of at the hydroxylated surfaces. Both Mn and Ti ions are reduced in response to vacancy formation.

Modification with Mn_4_O_x_ has a significant impact on the light absorption properties. Occupied Mn 3d states extend the VBM of the composites to higher energies relative to that of the titania support and empty states emerge below the CBM. The consequent red shift in the light absorption edge is confirmed by our model for the photoexcited state. In particular, the vertical energy, analogous to the optical energy gap, decreases significantly for Mn_4_O_5_-OH-a101 relative to that computed for the unmodified, hydroxylated anatase (101) surface. Analysis of this model shows that electrons and holes localize at Mn and neighboring O_C_ sites, respectively, so that modification may not promote separation of photoexcited charges, but the trapping energies of the electron and hole are quite high, suggesting high stability.

With regard to water adsorption and activation, the formation of oxygen vacancies has an impact on the strength of interaction and the most favorable adsorption mode of H_2_O at the modified surfaces. For Mn_4_O_5_-OH-a101, with a spontaneously formed O_V_, water adsorbs only in molecular form. With formation of reducing oxygen vacancies, water adsorption becomes more exothermic and leads to spontaneous dissociation to surface bound hydroxyls, similar to observations made for water interacting at reduced TiO_2_ (Schaub et al., 2001; Henderson et al., 2003) and CeO_2_ (Mullins et al., 2012) surfaces.

## Author Contributions

MN devised the research. SR performed the DFT modeling. Both authors analyzed the results and prepared, reviewed and approved the text of the paper.

### Conflict of Interest Statement

The authors declare that the research was conducted in the absence of any commercial or financial relationships that could be construed as a potential conflict of interest.
