# Supplementary material for: Activation of Water on MnOx-Nanocluster-Modified Rutile (110) and Anatase (101) TiO2 and the Role of Cation Reduction
Source: Front Chem. 2019 Feb 12;7:67. doi: 10.3389/fchem.2019.00067 (PMC6379279; doi:10.3389/fchem.2019.00067)
Supplement: Supplementary file 1 [file Data_Sheet_1.PDF]

Activation of water on MnO_x-nanocluster-modified rutile (110) and anatase (101) TiO_2 and the role of cation reduction.

Stephen Rhatigan and Michael Nolan*

Tyndall National Institute, University College Cork, Lee Maltings, Cork, Ireland

michael.nolan@tyndall.ie

Supporting Information

This document contains supplementary material relevant to the paper entitled: *"Activation of water on MnO_x-modified rutile (110) and anatase (101) TiO_2 and the role of cation reduction"*.

List of Figures:

S1: Relaxed atomic structure of the stoichiometric gas phase Mn_4O_6 nanocluster.

S2: Schematic diagram of the relationship between the energies computed in the photoexcited model.

S3: Relaxed atomic structures of Mn_4O_x-modified OH-r110 and OH-a101 with one and two oxygen vacancies. These structures are less stable but comparable in energy to those described in the main text.

S4: Computed DOS plots for unmodified, hydroxylated rutile (110) and anatase (101) and stoichiometric Mn_4O_6-OH-r110 and Mn_4O_6-OH-a101.

S5: Relaxed atomic structures for stable configurations of H_2O adsorbed at the reduced MnO_x-TiO_2 surfaces, including the computed adsorption energies.

METHODOLOGY

The following Figures and analysis are relevant to the **Methodology** section of the main text.

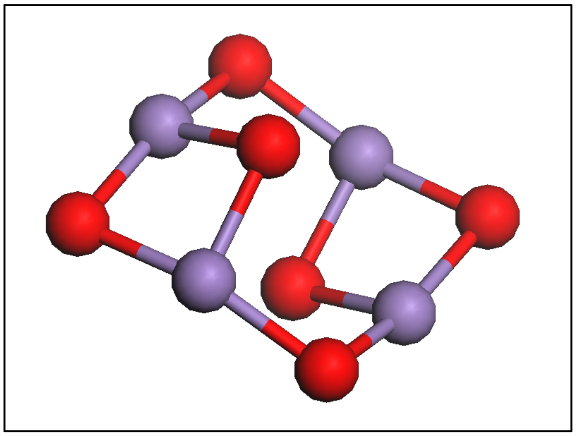

Figure S1 Relaxed atomic structure of the stoichiometric gas phase Mn_4O_6 nanocluster.

The Mn_4O_6 nanocluster modifier shown in Figure S1, was relaxed in the gas phase within the same computational setup described in the main text, with no constraints on the ionic positions.

The nanocluster was then adsorbed at the hydroxylated rutile (110) and anatase (101) surfaces in different configurations and each of these were relaxed, as described in previous work.[1-7] The most stable Mn_4O_6-OH-r110 and Mn_4O_6-OH-a101 heterostructures were used in the subsequent calculations. Although there are many possible adsorption structures of the nanoclusters on the titania surfaces, with a range of adsorption energies, we find that once the nanoclusters are adsorbed in stable configurations, the trends in key properties, such as band gap reduction are unaffected.[7]

Photoexcitation is modelled by imposing a triplet electronic state on the system to promote an electron to the conduction band, with a corresponding hole in the valence band. This model examines the energetics and charge localization associated with photoexcitation. The following energies are computed:

- The ground state energy of the system, yielding $E^{singlet}$.
- A single point energy calculation of the triplet electronic state at the ground state geometry, which gives $E^{unrelaxed}$.
- The fully relaxed triplet electronic state which gives $E^{relaxed}$.

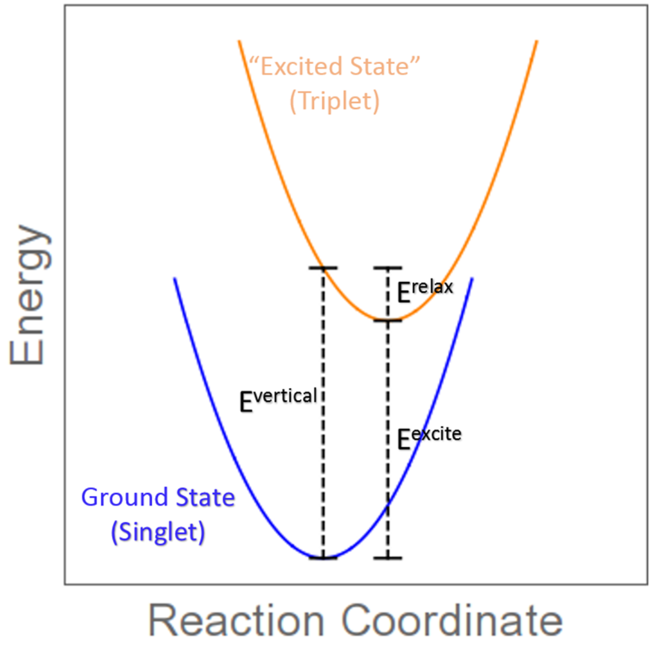

Figure S2 Schematic diagram of the relationship between the energies computed in the photoexcited model.

From the results of these calculations we compute:

1. The singlet-triplet vertical excitation energy:

$$E^{vertical} = E^{unrelaxed} - E^{singlet}.$$

   This is the difference in energy between the ground (singlet) state and the imposed triplet state at the singlet geometry and corresponds to the simple VB-CB energy gap from the computed density of states.

2. The singlet-triplet excitation energy:

$$E^{excite} = E^{relaxed} - E^{singlet}.$$

   This is the difference in energy between the relaxed triplet state and the relaxed singlet state and gives a crude approximation of the excitation energy.

3. The triplet relaxation (carrier trapping) energy:

$$E^{relax} = E^{unrelaxed} - E^{relaxed}.$$

   This difference in energy between the unrelaxed and relaxed triplet states is the energy gained when the electron and hole are trapped at their metal and oxygen sites upon structural relaxation. This energy relates to the stability of the trapped electron and hole.

These quantities are summarized schematically in Figure S2.

RESULTS

The following Figure and analysis are relevant to the **Section** of the main text entitled: ***"Reduction of Mn_4O_6-modified TiO_2 OH-rutile (110) and OH-anatase (101) via oxygen vacancy formation."***

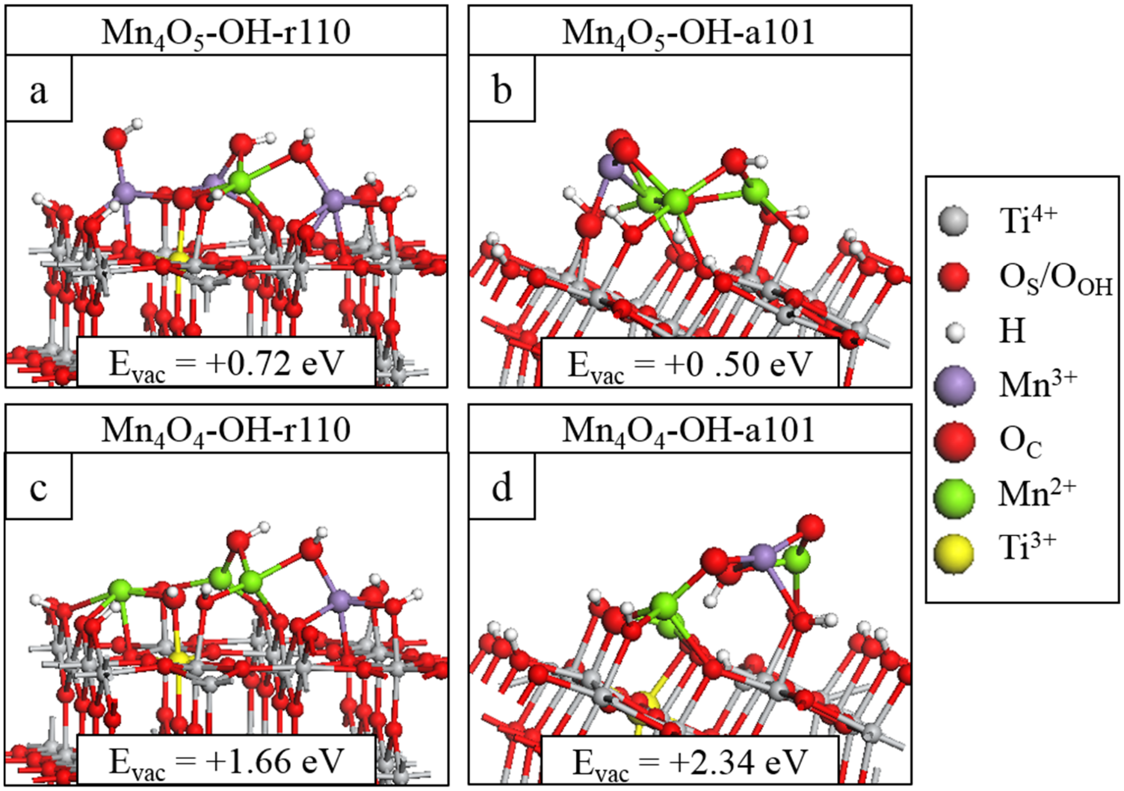

Figure S3 Relaxed atomic structures of additional configurations of MnO_x-modified titania surfaces with oxygen vacancies. **(a)** Mn_4O_5-OH-r110, **(b)** Mn_4O_5-OH-a101, **(c)** Mn_4O_4-OH-r110 and **(d)** Mn_4O_4-OH-a101. The formation energies are computed relative to the most stable structure with one less O_V.

The structures shown in Figure S3, their formation energies and distribution of ions should be compared with those shown in Figure 1 of the main text. For the configurations with one O_V, the structures described in the main text are more stable by ~0.9 eV than those shown in Figure S3(a) and S3(b). For the configurations with two O_V the formation energies are comparable and one would expect that Mn and Ti ions are present in a variety of oxidation states at the MnO_x-modified titania surfaces.

The following Figure and analysis are relevant to the **Section** of the main text entitle: ***"Electronic properties of Mn_4O_x-modified TiO_2 OH-rutile (110) and OH-anatase (101)."***

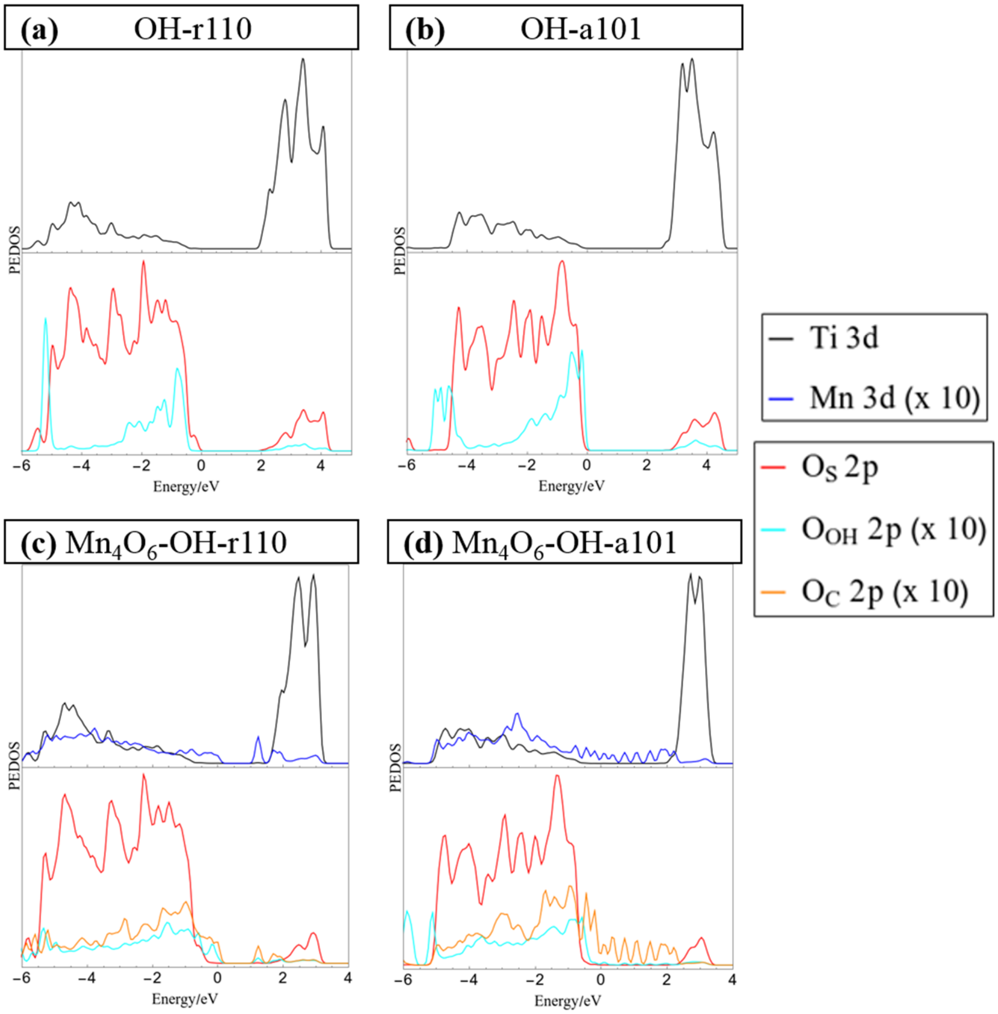

Figure S4 Computed DOS plots for unmodified, hydroxylated rutile (110) and anatase (101), and the stoichiometric Mn_4O_6-OH-r110 and Mn_4O_6-OH-a101 composites.

From Figure S3 (a) we can see that for unmodified rutile (110) that the presence of hydroxyls has no impact on the bandgap; the highest occupied hydroxyl-derived states lie lower in energy than the VBM of the rutile (110) surface. For unmodified anatase (101), the hydroxyl-derived states overlap the O 2p states of the surface at the valence band edge. From these plots we can conclude that the computed bandgaps are *ca.* 2 eV and 2.7 eV for hydroxylated rutile (110) and anatase (101), respectively; this agrees with analysis of the photoexcited model applied to these systems and described in the main text.

For Mn_4O_6-OH-r110, shown in Figure S3(c), nanocluster derived states extend to 0.3 eV above the VBM of the rutile (110) surface. Unoccupied Mn 3d-derived states also emerge in the titania band gap at 0.4 eV below the CBM. For Mn_4O_6-OH-a101, the modifier-derived states span the titania bandgap. However, these systems favours non-stoichiometry as O_V form spontaneously and so are present in the

ground state as Mn_4O_5-OH-r110 and Mn_4O_5-OH-a101 (see main text). We may conclude that these computed DOS do not represent a physical system and they have been included here for completeness.

The following Figures and analysis are relevant to the **Section** of the main text entitled: ***"H₂O adsorption at Mn₄Oₓ-modified OH-rutile (110) and OH-anatase (101)."***

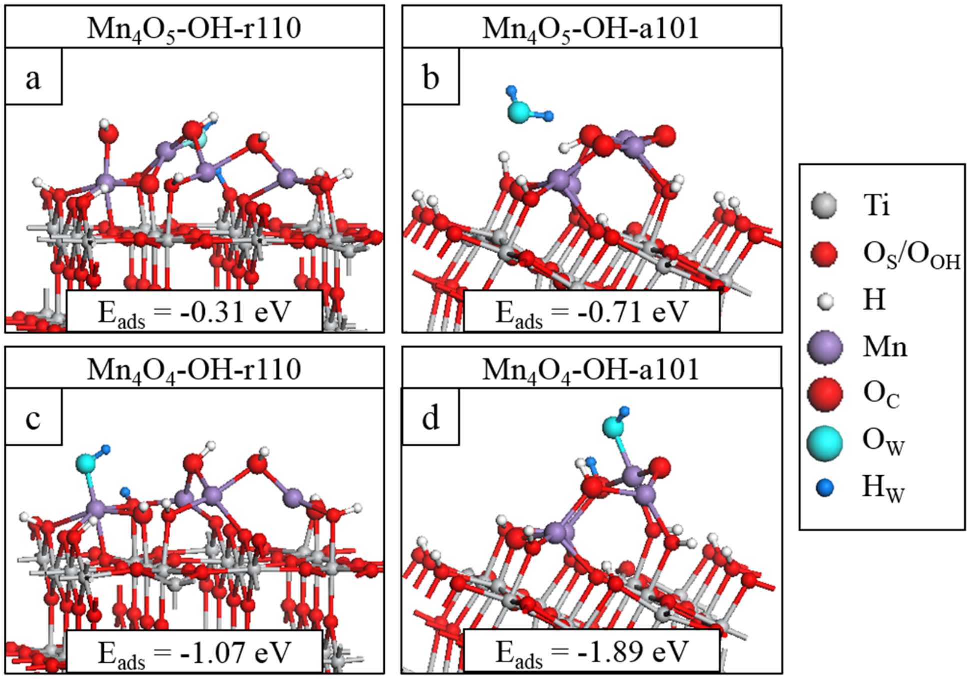

Figure S5 Relaxed atomic structures for stable configurations of H_2O adsorbed at **(a)** Mn_4O_5-OH-r110, **(b)** Mn_4O_5-OH-a101, **(c)** Mn_4O_4-OH-r110 and **(d)** Mn_4O_4-OH-a101. Atomic species are distinguished by colour according to the legend on the right hand side.

Figure S5 displays additional stable configuration for water molecules adsorbed at the off-stoichiometric Mn_2O_x-modified titania surfaces. For the structures with a single O_V, water only adsorbed at the vacancy site and did so both molecularly and dissociatively for Mn_4O_5OH-r110 and only molecularly for Mn_4O_5OH-a101. The final relaxed geometry and adsorption energy differed depending on the initial adsorption set-up and the most stable configurations are described in the main text. For the modified surfaces with two O_V, water adsorption was favourable at multiple sites and led to spontaneous dissociation. The interaction is strongest for water adsorbed at Mn_4O_4-OH-a101; computed adsorption energies were in the range of -2.0 eV to -1.3 eV.

REFERENCES

1. M. Fronzi and M. Nolan, *ACS Omega*, 2017, **2**, 6795-6808.
2. M. Nolan, A. Iwaszuk, A. K. Lucid, J. J. Carey and M. Fronzi, *Adv. Mater.*, 2016, **28**, 5425-5446.
3. A. Iwaszuk and M. Nolan, *Phys. Chem. Chem. Phys.*, 2011, **13**, 4963-4973.
4. A. Lucid, A. Iwaszuk and M. Nolan, *Mater. Sci. Semicond. Process.*, 2014, **25**, 59-67.
5. A. Iwaszuk, P. A. Mulheran and M. Nolan, *J. Mater. Chem. A*, 2013, **1**, 2515-2525.
6. M. Fronzi, A. Iwaszuk, A. Lucid and M. Nolan, *J. Phys.: Condens. Matter*, 2016, **28**, 074006.
7. M. Fronzi, W. Daly and M. Nolan, *Appl. Catal., A:* , 2016, **521**, 240-249.
